# Parks and Safety: How Cognitive Decline May Impact Access and Perception

**DOI:** 10.1093/geroni/igaa057.2473

**Published:** 2020-12-16

**Authors:** Philippa Clarke, Michael Esposito, Joy Bohyun Jang, Sandra Tang, Anam Khan, Dominique Sylvers, Jessica Finlay

**Affiliations:** University of Michigan, Ann Arbor, Michigan, United States

## Abstract

Older adults’ perceptions of the presence and quality of neighborhood resources provide important information about the potential benefit of those resources but are not necessarily concordant with the actual physical resources available in that environment. There is debate about whether subjective perceptions of local context are more important for individual behavior and well-being than objective indicators of resources. However, little research has examined how cognitive function is related to differences in the perceived availability and quality of neighborhood resources among older adults. We found that subjective reports of neighborhood safety and adequacy of parks were positively associated with objective measures of property crime and park density. Cognitive function was associated with higher subjective neighborhood evaluations, but adults with lower cognitive function reported more discordance between objective and subjective measures of neighborhood resources. These findings inform how neighborhood resources may have different consequences for older adults experiencing cognitive decline.

